# Eye-tracking and learning experience: gaze trajectories to better understand the behavior of memorial visitors

**DOI:** 10.16910/jemr.13.2.3

**Published:** 2020-05-16

**Authors:** Salma Mesmoudi, Stanislas Hommet, Denis Peschanski

**Affiliations:** CNRS, Univ. Paris 1 Panthéon-Sorbonne, EHESS, HESAM Univ., UMR8209, CESSP, MATRICE, France; MATRICE, France

**Keywords:** eye movement, eye-tracking, saccades, gaze trajectories, memorial, attention, text-mining, focus group, museum

## Abstract

Eye-tracking technology is increasingly introduced in museums to assess their role in learning and knowledge transfer. However, their use provide limited quantitative and/or qualitative measures such as viewing time and/or gaze trajectory on an isolated object or image (Region of Interest "ROI").The aim of this work is to evaluate the potential of the mobile eye-tracking to quantify the students’ experience and behaviors through their visit of the "Genocide and mass violence" area of the Caen memorial. In this study, we collected eye-tracking data from 17 students during their visit to the memorial. In addition, all visitors filled out a questionnaire before the visit, and a focus group was conducted before and after the visit. The first results of this study allowed us to analyze the viewing time spent by each visitor in front of 19-selected ROIs, and some of their specific sub-parts. The other important result was the reconstruction of the gaze trajectory through these ROIs. Our global trajectory approach allowed to complete the information obtained from an isolated ROI, and to identify some behaviors such as avoidance. Clustering analysis revealed some typical trajectories performed by specific sub-groups. The eye-tracking results were consolidated by the participants' answers during the focus group.

## Introduction

In an empirical way, Caen memorial-museum officials noted that, among the artifacts displayed throughout the exhibition space, the visitors looked at much more ima-ges and objects, especially animated images, than texts. They also confirmed that the recorded testimonies attract a lot of attention. However, even though these fixed or animated images could be emotional attraction materials, it is clear that the visitor, especially when students, tend to read historical commentaries more as a means of verifying or confirming and completing what they learn in class.

In order to understand and evaluate memorial-museums as vectors of new learning and/or reinforcements of prior knowledge [1, 2, 3, 4, 5], several methods were used to quantify the visitor experience. In this context, Packer et al. [6] designed and developed an instrument to capture multiple facets of the visitor experience using a simple and unobtrusive adjective checklist. More precisely, for the dark tourism, that involves a visit to real or recreated places associated with death, suffering, misfortune, or the seemingly macabre, Nawijn et al. [7, 8, 9] tried to capture emotional feelings generated during visit to a concentration camp memorial site Whereas, other researchers [10, 11] worked on the motivation to visit these dark destinations.

Research based on walk-along method, focuses on spatial practice, personal biographies and social architecture among visitors were performed to understand the visitor experience [12]. The walk-long method was described as a hybrid between participant observation and individual interviews [13]. Another way to describe the visitor experience is the focus group [14, 15, 16]. This qualitative method takes the form of in-depth discussions with groups of about eight to twelve participants, lasting from one to two hours. Focus groups are led by a well-trained discussion moderator who follows a guideline with topics, questions, probes, and target timings. Participants are encouraged to stick to the discussion topic but to express whatever is on their minds. This loosely structured format allows identifying the range of audience reactions, attitudes, issues, expectations, and perceptions related to the topic.

These tools provide quantitative or qualitative understanding of the visit meaning, but they do not provide an understanding of what exactly happens when the visitor is looking at the work or image in the dialectical interaction between visitor and objects or images seen during the visit.

In fact, when facing an object, eye movements can be of different types: fixations (when the eyes are relatively motionless), pursuits (when the eyes follow a moving target) or saccades (rapid movements between fixations). Some studies suggest that fixations and saccades indicate how people acquire information [17, 18]. Specifically, saccades may indicate the emotional state of the participants when they evocated emotional autobiographical memories [19]. Furthermore, participants of Nummenmaa et al. [20] study, performed vertical reflexive saccades that were orthogonal to the emotional-neutral picture locations. Saccade endpoints and trajectories deviated away from the visual field in which the emotional scenes were presented.

To quantify these visual interactions, some studies have focused on the observation of visitors and the calculus of Viewing Time (VT). Smith and Smith [21] manually timed this VT on six paintings in the permanent collection of the Metropolitan Museum of Art in New York, which contains more than a million objects. The introduction of the eye-tracking in the museum studies [22, 23, 24, 25, 26, 27, 28, 29, 30, 31, 32] made the calculus of VT and saccades much more precise.

However, despite all the clarifications that eye-tracking can provide a verbalization of visitors' thoughts, called also a retrospective [33], could shed light on the true behavior of these visitors and avoid over-interpretation of these eye movements.

To perform our study, we used a hybrid method that combines eye-tracking and focus groups.

The aim of our work is to evaluate the potential of the mobile eye-tracking to quantify the visitors experience and behaviors of student’s group through their visit of the "Genocide and mass violence" area of the Caen memo-rial-museum. In addition, focus groups were held before and after the visit to provide additional keys to analyze eye tracking data. Precisely, the correspondence between the typical behaviors detected by eye-tracking and the two forms of learning: assimilation and accommodation.

Our approach will provide answers to some of the major questions raised by the visit to memorials, such as the time actually spent on the different items, what is actually looked at in the item in question, possible typical behaviors that therefore go beyond the visitor's individuality, and the preferred itineraries in the routes at different scales.

## Methods

### Participants

Seventeen students (age range = 17-18 years; Males/Females = 4/13) were recruited. Thirteen participants were senior high school literary students and 4 were senior high school science students. Participants came from two different schools, one located in the city centre of Caen (Lycée Victor Hugo), the other in Honfleur (Lycée Albert-Sorel). In French high school, the Second World War is studied during the second to penultimate year («Première») in an a-chronological order that has long surprised historians and will be questioned in the new curricula. Indeed, the two World Wars are taught first, then is totalitarianism (Nazism and Stalinism), while it was not until the end of the school year that France in the dark years, especially the collaborating French state based in Vichy, was treated. The 17 participants in our panel were in their final year of high school, where the beginning of the year is devoted to the study of the memory of Vichy. To prepare for the visit to the memorial-museum, the teachers presented extracts from television news reports on the commemorations of the Velodrome d'Hiver Rafle in 1992 and 1995, in the presence for the first time of the President of the Republic, François Mitterrand in 1992, and of his successor Jacques Chirac in 1995. In a speech that had a great impact, Jacques Chirac insisted on the responsibility of France at a State level and not only of a sum of individual collaborators in the deportation of Jews. It was the first time this was said at the level of a President of the Republic.

#### The visit scenario and Regions of Interest.

Since its opening, the Caen memorial (400,000 visitors per year on average) is mainly dedicated to the history of the Second World War since its opening (1988). It is the most visited museum about Second World War in France, and is regularly updated to take account of advances in historiography.

The room devoted to "Genocide and mass violence" is the most important in the "World War, Total War" area. Covering some 400 m^2^ in total, the room evokes the massacres that took place between 1937 and 1945 in Europe and Asia.

The reference route begins after the introductory sequence called "The extermination of the Jews in Europe" on the map. The sequence mainly shows Hitler's speech of January 30, 1939, where he announces that in the event of a new world war, the Jews would be exterminated. The space is divided into 7 thematic areas (see details of the objects and pictures exhibited in Supplementary Material "SM1").

- Zone 1: "The first steps: the T4 euthanasia program" for the mentally ill. In yellow on the map, there is an introductory text that provides the historical content, as well as a photograph and a poster.

- Zone 2: "From persecution to extermination". Dark green on the map is devoted to the various forms of humiliation, stigmatization and exclusion of Jews before their deportation and extermination.

- Zones 3-4: "The extermination of proximity" is devoted to the so-called "Holocaust by bullets" in the territories of Eastern Europe, accompanying the German offensive against the Soviet Union. It is a very specific space of the memorial: images, objects and testimonies provided by Father Patrick Desbois and his association Yahad-in-Unum present at length a policy of extermination that the general public is not familiar with.

- Zone 5: In purple on the map, the area concerning “ghettos” is located in a corner of the exhibition. Two videos are proposed to the eye, they are accompanied by a short cartel.

- Zone 6: "Killing centers". Of different colors on the map, it has been divided into 5 sub-zones. 1. The extermination process. 2. The Kirzners. 3. The fate of the children. 4. The Sonderkommandos. 5. The camps

-Zone 7: "Gypsies". In emerald green on the map, it shows the fate of the Gypsies in the Reich. Again, this is a subject that is hardly ever found in the museums of Europe devoted to the Second World War or in school curricula.

The designers conceived the exhibition so that, even without visible limits in space, the visitors go from one area to another in the order presented here.

**Figure 1.**
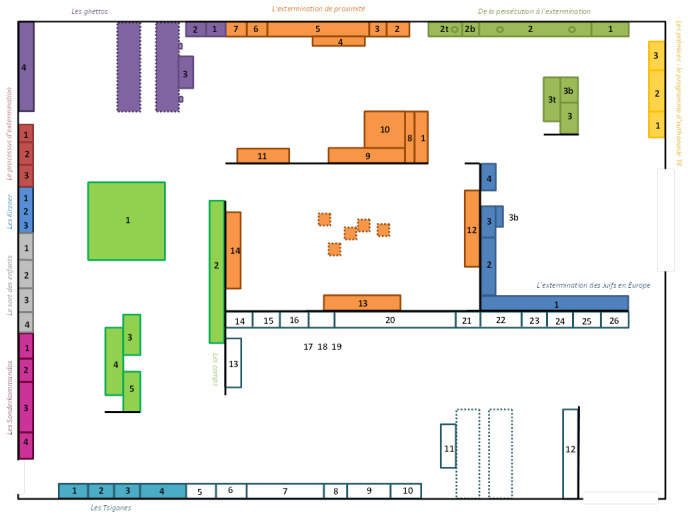
Plan of the "Genocide and mass violence" room. The details of each element are in the plan is in SM1.

An introductory panel is positioned at the beginning of each area, placed against the wall. The white writing on a black background singularizes it and thus indicates, each time, a historical presentation of the subject. The exhibits objects, or images, are accompanied by cartels that are generally located below or to their side. The written testimonies are presented in white letters on a red background. Finally, it should be noted that the space is uniform, with free circulation, without constraints, with no steps or ascents or descents that could mark a break between the zones.

### Design

In this study, three primary data were provided. We extracted fixations’ coordinates as well as their start and end time from the eye tracking data. We consider the third primary data to be the verbatim extracted from focus groups that were held before and after the visit.

**Figure 2.**
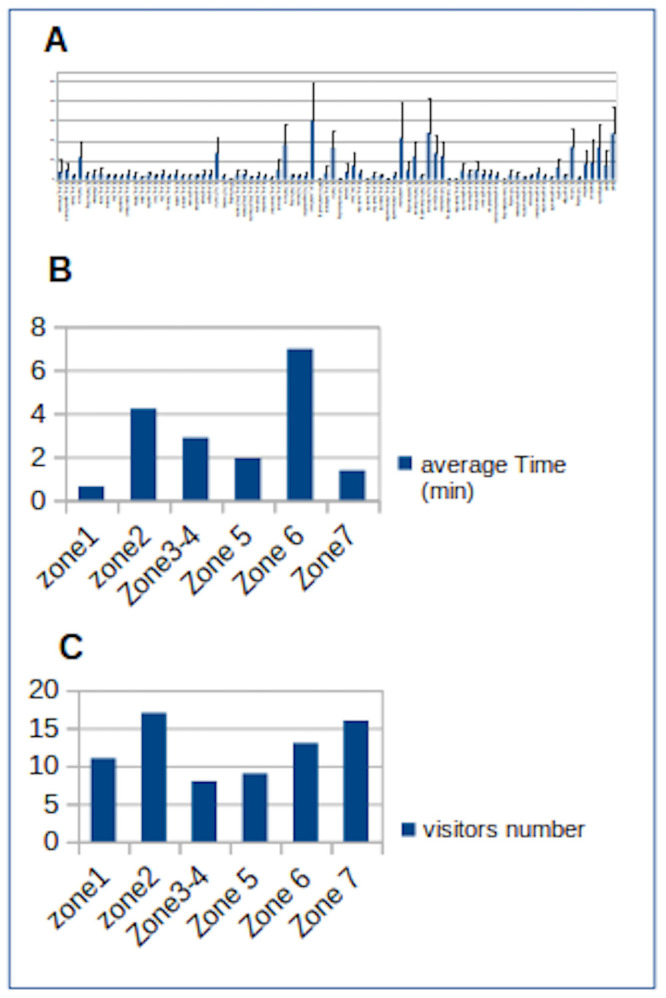
A. Graph of average time spent in each region of interest (ROI) in seconds with standard deviation (table of values is in supplementary material SM2). B. The average time in minutes spent by participants in each area. C. The maximum number of visitors in front of an item in each zone.

The first variable that we presented was the viewing time (VT). In the Figure 2 we presented averages with standard deviations.

From the fixations' coordinates and their order, we reconstructed local trajectories in one "ROI", then we mapped:

- all the gaze path of participants for the drawing image of "oven"

- an example of the gaze path of one participant named (E11), over the letter of a Waffen SS.

For global trajectory through several "ROIs", we presented two examples, the wall of photographs (second zone) and the photographs of the Mizosz massacre. From the first example, we provided also the denser paths and saccades.

Finally, with the extracted verbatim, we calculated the number of occurrences of each term from the transcripts of the focus groups.

### Materials

#### Eye-tracking.

The 17 students all wore double-focus glasses of the ASL brand (version 2.02), from Imotions company (Human Behavior Research). We choose this device because it is non-intrusive. It does not require electronic sensors to be placed in the paths at the four corners of each item or, in the form of a bar, underneath the series over two or three meters. The memorial-museum where our study was carried out is very attached to the aesthetic constraint of the device.

Moreover, thanks to the ASL Results+ GM program, we were able to get the coordinates of the fixing points and the durations.

#### ASL detection of fixation and saccades

According to the ASL MANUAL VERSION 2.02 [34], the fixation algorithm derives from work done by Lambert [35], and further developed by Flagg [ 36] and Karsh & Breitenbach [37]. It has been further developed at ASL over the years. The method falls in the category that Duchowski [38] labels “dwell-time fixation detection”.

Based on the minimum time needed by the nervous system to process visual information meaningfully, and therefore the shortest sensible “snapshot”, the first studies range the shortest latencies between 100 ms and 300 ms [39, 40, 41]. Looking at more recent data, saccadic latencies seem rarely to be less than 150 ms under most conditions, and are more typically over 200 ms, but “express saccades” can have latencies as short as 90 to 120 ms when the old fixation target disappears before the new target appears, or if the new targets are predictable [42, 43].

The default “minimum” in the ASL fixation program is 100ms. Note that if the data is collected at 60 fields per second, it corresponds to 7 samples. The 1-degree minimum change in gaze position required to define a new fixation is based, loosely, on the fact that miniature eye movements (tremor, drift, and micro-saccades) are generally smaller than 1 degree.

#### Visual angle computations

To retrieve the eye-tracking results in the form of fixation times and coordinates on a fixation plane, we therefore used ASL Results Plus GM analysis software.

According to the ASL Manual, all fixation criteria are defined in degrees of visual angle, i.e. how much eye turns between measured points of gaze. Therefore, we needed to translate point of gaze data expressed in eye tracker units (remote optics) or real distance units on a surface (head-mounted optics with EyeHead Integration) to degrees.

If we assume that lines of gaze are more or less perpendicular to the surface (within about 20 degrees), visual angle A between two points is defined by the equation:

tan (A) = D / S

where D is the distance between the points, and S is the distance from the eye to the scene plane.

In order to avoid time consuming calculation of tan -1 we use the fact that for fixation analysis we are only interested in small eye movements and we use the small angle approximation:

tan (A) ≅ A,

where A is expressed in radians.

There are 180/pi (=57.2958) radians per degree. Combining the two equations and translating radians to degrees we get the equation that the analysis program uses to calculate visual angles:

A = 57.2958 * D / S

where A is the visual angle in degrees, between two points separated by distance D, at a distance S from the eye.

The analysis program takes the difference between points of gaze coordinates and divides by the user specified constant labeled “Eye tracker units/degree”. The default value is 10, which is roughly typical for a table-mounted up.

#### Focus groups:

The aim of the focus group was to highlight the way these young people consider the Holocaust: their appropriation of ideas, the negotiations to develop them, their points of agreement or disagreement, the sometimes mutual incomprehension, which leads group members to accept or refuse such or such argument.

The project did not seek to verify the academic knowledge of the young people, but rather their capacity to understand debate and invest the subject with their own values and emotions.

During the focus groups (before and after the visit), the questions asked dealt with the following themes:

- the study of the variety of opinions and feelings of the actors on a given subject,

- highlighting differences in perspective between groups of individuals,

- understanding of the results of school learning and possibly how students viewed the course on the Holocaust

- the appropriation of historical knowledge in a museum.

The sessions lasted from 55 to 70 minutes maximum. Both groups were interviewed in their respective schools before the visit and then in a room provided by the Memorial at the end of the visit.

The focus groups took place without the presence of teachers from the school. The sessions were recorded and transcribed manually afterwards.

#### Statistical strategy

As already mentioned, the software that comes with the ASL eye-tracking glasses allows to obtain, upstream of the heatmaps and graphs built by the tool itself, the coordinates of the fixations and the fixing time of each coordinate. For the sake of accuracy and verification and to complete the results, we have recalculated all the histograms and eye trajectories for each participant and for each region of interest (ROI).

From these calculations we were first able to obtain clusters from the fixations, so that we could calculate the exact gaze duration on each element within a region of interest. We were then able to reconstruct trajectories that connect several regions of interest. This was extremely important in so far as it enabled us to follow the gaze as it travelled across a significantly large area.

All of the scientific calculations performed were developed using the R cran programming language [44].

#### Trajectory density detection algorithm:

To perform common sub part of gaze trajectories obtained from all 17 participants, we used a TRAjectory CLUStering algorithm TRACLUS [45]. This algorithm has two phases: partitioning and grouping. The first phase is based on the trajectory partitioning algorithm using the minimum description length (MDL) principle. The second phase of algorithm uses a density-based line-segment clustering algorithm. We used the python-programmed version of this algorithm, which is deposited in github (https://github.com/apolcyn).

#### Text-mining on focus groups:

In order to analyze more precisely the focus groups performed before and after the visit, and transcribed manually, we used the lexical extraction capabilities of the Natural Language Toolkit (NLTK) library in python [46]. The NLTK is a suite of open source program modules, tutorials and program sets, which covers symbolic and statistical processing of natural language and is interfaced to annotated corpora.

### Procedure

Our study, which took place in the "Genocide and mass violence" room of the Caen memorial-museum, was carried out in the following stages:

1. The students watched two extracts from television news reports on the commemorations of the Velodrome d'Hiver Rafle in 1992 and 1995, in the presence for the first time of the President of the Republic, François Mitterrand in 1992, and of his successor Jacques Chirac in 1995.

2. focus groups were held in the classrooms of the two schools of the participants, without the presence of their teachers.

3. the 17 participants wore the ASL glasses during a visit of the space under study.

4. At the end of the visit, focus groups were held in the memorial-museum, without the presence of the teachers.

5. The data were retrieved from the ASL computer and processed later in the laboratory.

## Results

In this work, we relied mainly on eye tracking data. The verbatim of the focus groups provided clarification on the results obtained, but the main point of this article is to present the results of the eye tracking analysis.

From the eye-tracking data, we were able to obtain several types of results ranging from the global quantification of the time spent in each area of the visit space to the time spent in front of each Region of Interest (ROI) by each participant. The same data allowed us to reconstruct the trajectory of each participant's gaze in front of a region of interest and/or a set of regions of interest.

### Viewing time (VT)

One of our first results is the average time spent in the space by our participants: namely 20'14''. Students spent on average 3'93 in the introductory part and moving around in the space between the zones. Our analysis focuses on the time spent on the different zones on the map. Eleven students spent less than 20'14 in our observation zone and 6 more than 20'14. The visit of four students lasted longer than 30 minutes. This average visiting time is higher than what the museum identifies as the « usual time » for adults in the observed space (15 minutes).

None of the students looked at all of the exhibits in each zone, and only "The Wall of Photographs" in Zone 2 was looked at by all 17 students.

#### Quantification of time spent in front of each region of interest

The region of interest (ROI) can be an image, an object or a text, which is not without consequence on the time spent in front of each one. One may imagine that a text calls for a longer time of attention than an object or an image. Yet, museum officials have long observed that visitors spend less time on the historical commentary than on the image, the object or the moving image – an attitude that could be linked to the fact visitors to memorials think they already know the content of the commentary.

As shown in Figure 2, the average time spent in front of the ROI of each area varies between 0.2 and 29 seconds. The standard deviation is very large in such averages because some people spent more than a minute in front of a ROI and others much less than a second.

On the other hand, according to the same figure, we see that the ROI our group looked at the longest is the letter written by the Waffen SS officer to his wife, located in zone 3. The other ROI where the participants spent the most time are either texts or captions of the pictures.

Another equally interesting result in our work is the number of visitors who stop to read the texts or image titles, from 11 to 16 participants which corresponds to 65% - 94% of our sample.

Of course, it is not possible to compare zones of varying length. It is however important to note that only the wall of photographs illustrating the mechanisms of humiliation and exclusion, "From Persecution to Extermination", was viewed by all participants. Secondly, we note that zone 7 dedicated to "Gypsies" is both the one that was seen by the greatest number and the one where the time spent is the shortest.

As an example, we will focus on the most striking exhibits:

- Written testimonies: two letters on extermination - one on the extermination of proximity, the other on the crematorium of Auschwitz 2.

- Photographs: the very peculiar wall on the persecution before deportation, about fifteen photographs considered as a single depot, and period photographs of the Mizocz massacre.

#### Quantification of the amount of time each participant spent looking at each part of an area of interest.

**Figure 3.**
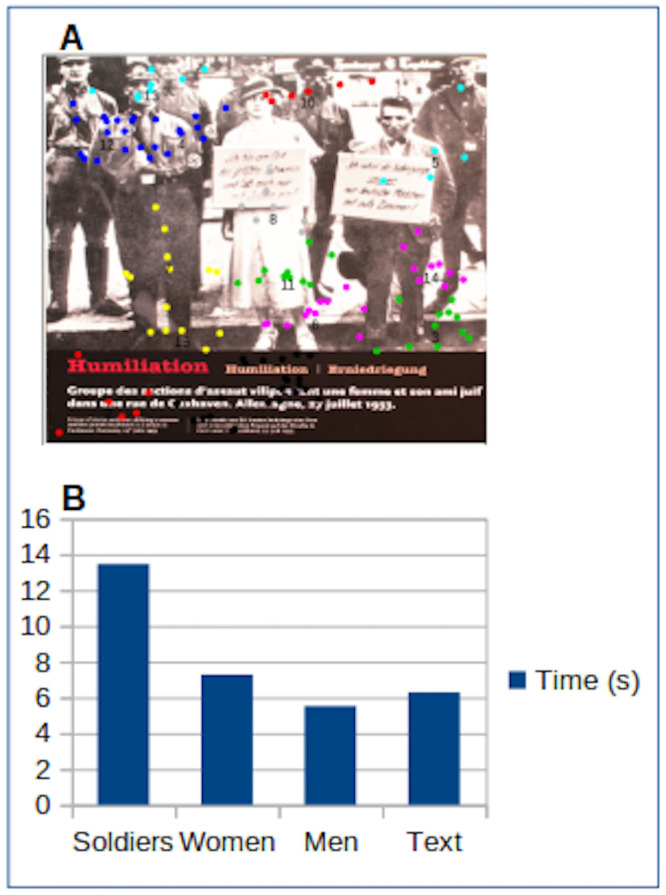
A. Projection of the fixation coordinates of the different participants who looked at this image, each cluster being identified by a color. B. Graph of the time of fixations in seconds for thematic clusters.

We were able to project the coordinates of the fixations of all participants on the image looked at (ROI), thanks to our clustering algorithm. We then identified clusters of fixations; each group is represented by a different color (see Figure 3). From these clusters, we identified the position of each point; we then calculated the duration of the fixation associated to this point.

From the graph in Figure 3B, we could deduce that the SA (Sturmabteilung) was the most "looked at" by the participants. The figure that comes next is the woman whilst the man on the picture is the less “looked at”. Some participants also tried to read the stigmatizing signs worn by the couple. Yet undoubtedly, the majority of the gaze focused on the SAs to the right of the woman. One possible observation is that the perpetrators are observed more than the victims.

### Gaze trajectory

#### Gaze trajectory at the scale of a ROI

A gaze trajectory could be traced from the coordinates of the fixations and their order.

*A. mapping of one gaze trajectory (participant E11) on/ a letter written by a Waffen SS officer:*

This letter was written two days after the massacre of Jews in which the officer took part, often caught the attention of the students. The emotional charge is very important, which is not without consequence on the reading.

11 of the 17 participants looked at this letter with great attention (30 seconds on average).

**Figure 4.**
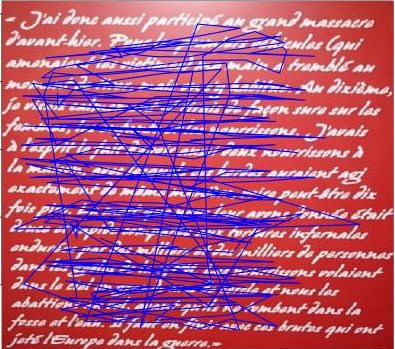
A participant's gaze trajectory from the fixation points on a letter sent by a Waffen SS to his wife.

In the reading example shown in Figure 4, the participant reads the text twice, dedicating 1'47 min to it. The reader jumps from the caption to the beginning of the letter, hence the first vertical line that cuts through the text. Then the student’s gaze had been relatively focused on the words, line by line, it was from the reference to "infants" that everything became blurred, the reader's gaze proceeded in a succession of saccades. The student returned to what he had previously read and went back and forth in the text (vertical saccades).

In this testimony, the Waffen SS explains that he participated in a massacre in the context of what is now called "the Holocaust by bullets" [47]. He further details that he was led to kill old people, women and children and that he shot infants thrown into the air by his comrades. Killing infants explained this SS was indispensable because, otherwise, they would come and kill more of their children and grandchildren in revenge.

The sentences that most appealed to the students are circled in yellow: "I was calmly aiming and safely shooting at women, children and infants," reads the first case. In the second, the gaze was fixed on "torture", "infants were flying in the sky".

All the participants who read this letter were marked by its content, as confirmed by the focus groups after the visit, but this is translated differently: there is the participant who reread the text in a linear way after having made many saccades during the first passage, the one who read quickly and looked for the end of the story from the sixth line. Only one participant apparently showed no emotion by normal horizontal reading.

*B. mapping of all participants’ gaze trajectories on the picture drawing by a survivor of the Sonderkommando, David Olère:*

The drawing details the work to which he had been assigned in crematorium III at Auschwitz-Birkenau. In this process of extermination wanted by the Nazis, which combined the gas chamber and crematorium, secrecy was of major importance. The drawings from inside the crematoria were made with great precision by David Olère immediately after his release, in order to bear witness to the horror.

**Figure 5.**
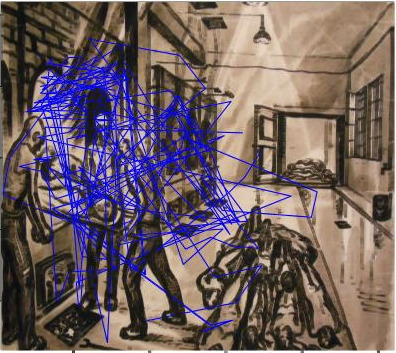
Gaze trajectories of all the participants who looked at this image of a crematorium.

In this picture (see Figure 5) we follow the eyes of the students. The paths have been reconstructed from the coordinates of each person's bindings.

It is noticeable that the gaze is focused on the ovens themselves and especially on the two members of the Sonderkommando who put a body in the oven, much more than on the pile of bodies at their feet or on the door at the back of the room, where a freight elevator brings its share of corpses from the gas chamber.

#### Gaze trajectory through a set of images or ROI and detection of saccades

*A. Analysis of the wall of photographs.*

The area studied is located in zone 2, "From persecution to extermination" (see Figure 1).

**Figure 6.**
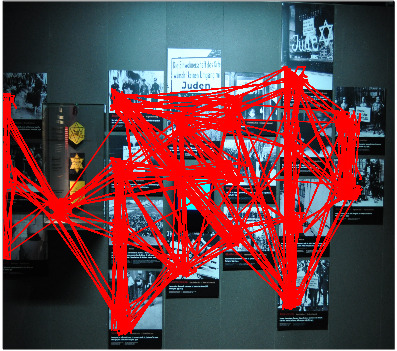
Mapping of all the trajectories of the 17 participants going through the wall of photographs in zone 2, made up of 19 ROI.

The students were confronted with this wall, which contains a set of photographs and three video screens. They spent a certain amount of time, with the average for the group being 4'22'', with significant variations ranging from 1'10'' to 12'. In Figure 6, we are mapping all the gaze paths of the 17 participants.

The gaze explores the wall of pictures in sense of the visit, from the right. The gaze first goes through one of the pictures of the first column. Both are systematically seen.

After analyzing each gaze trajectory mapped in Figure 6, we noticed that the students look at all the images. Only one student is an exception. He starts with the image of the old man dying on a ghetto pavement (2nd column) and then quickly leaves the right-hand columns.

In order to detect automatically typical sub-trajectories, we used the TRACLUS algorithm, which detects gaze densities from all participants’ fixations.

**Figure 7.**
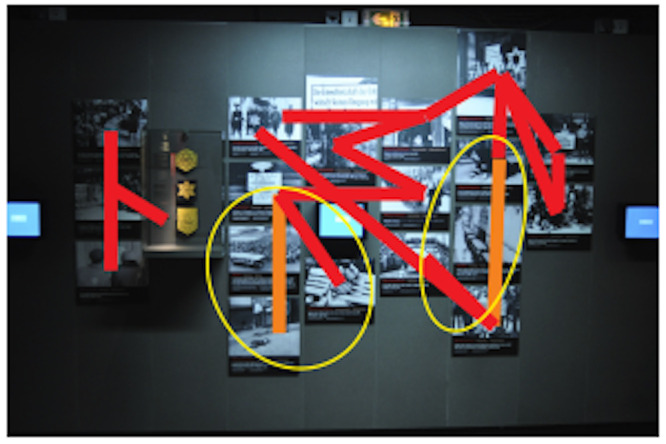
Clustering result on the trajectories of all the participants. The segments show the densest parts of trajectories. In orange, we find the densest segments but which, in addition, experience strong saccades.

In Figure 7, red and orange indicate the path segments with the highest density viewings.

In contrast to the red segments which materialize a one-way passage of the eye, the orange segments indicate saccades. The participants went back and forth vertically between these images.

We note that the students made the same associations between some of the images on the wall, such as the horizontal red bar at the top that groups together the pictures of stigmatization by the Jewish star. Other images are points of support that distribute the gaze. This is obvious for the two pictures on the right, the ones at the beginning, but it is also true for the one in the fifth column where a handwritten poster indicates, in German and French, "Juden unerwünscht" "Interdit aux Juifs". We can see that the participants return to this image to explore others.

*B. Analysis of the photographs of the Mizosz massacre*

It is this series of photographs taken by a German gendarme and showing the different stages of this massacre that is the most watched by the students.

They were seen by 14 of the 17 students with a VT of 20 to 30 seconds. The scenography of this part is important since the photographs are reduced in size and placed at a certain height in the wall which was dug out so that the pictures are distant from the eye of the visitor.

**Figure 8.**
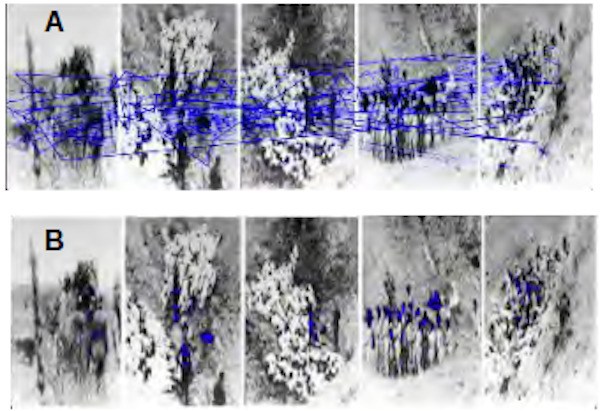
A. The gaze trajectories of 14 participants over a photo of the massacre of Ukrainian Jews in Mizocz, USSR. B. The densest points of fixation.

Figure 8.A shows the trajectories of these 14 students' gazes. From the fixations of participants mapped on the Figure 8.B, we notice that these fixations are denser on the heads of the victims compared to the heads of the officers in charge of killing them.

### Focus groups analysis by text-mining

After the museum visit, 9 students (out of 17) stated that the exhibition gave them more details, and that they acquired and/or were able to access new knowledge: "we learned more things we were able to improve our knowledge".

10 out of 17 participants expressed a questioning about the responsibility for the crime, but also about the capacity of man to commit such horrors.

A slightly more precise analysis of the verbatim allowed us to go further and question the relationship between teaching in the school setting and visiting memorials.

The first striking result is the comparative number of words used during focus groups: with 11985 before the visit against 22371 after the visit to the museum, the number has almost doubled.

**Figure 9.**
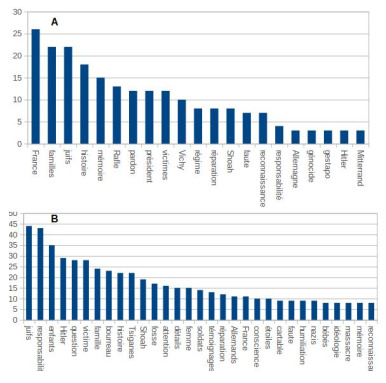
Graphs of the occurrences of the most used words in the participants' speech: A. during the class focus group. B. during the focus group at the end of the visit to the museum.

But, more interestingly, the hierarchy of words has been profoundly modified, even though in both cases the students are supposed to refer to the same period of time. Figure 9.A shows a polarity on the discourse on memory, on responsibility, on France, on the persecution of the Jews, and more precisely on the roundup of the *Velodrome d'Hiver*, namely the first and most important roundup organized on the initiative of the Germans, with the help of the Vichy collaborating regime, on July 16 and 17, 1942.

After the visit to the memorial (Figure 9.B), the persecution of the Jews is still central in the focus group's discourse, but at the heart of this process are the Germans, the Nazis, and Hitler. In addition, there is an overlapping of scales, from the main actors of history to the individual executioners and victims. For a complete list of the extracted words, see the supplementary material – SM3].

It is rare that the hierarchical list of occurrences alone is sufficient to distinguish so clearly between two discourses, and moreover between the same people on the same subject. It is not the purpose of this article to go into detail about the vocabulary used thanks to the classical tools of textometry. Such a blatant statement is enough, but what can it correspond to? We are in fact confronted with two different vectors of collective memory. The Caen memorial-museum is specifically intended to provide keys to understanding the Second World War. The space that was privileged for our study ("World War, Total War") shows the global dimension of the war, whereas the case of France is dealt with at length in the previous space. Drawing on the most recent historio-graphical advances, it focuses in particular, on the persecution of the Jews, on the mass massacres organized on the Eastern Marches by the Einsatzgruppen, the so-called "Holocaust by bullets". An important space is devoted to the other targets of Nazism, such as the Gypsies.

As explained in the section “Methods”, to prepare students for the visit to the memorial, the teachers presented extracts from François Mitterrand and Jacques Chirac speeches, to dig into the difficult sedimentation of education in schools from one year to the next.

## Discussion

In this work we focused on 3 results, the VT, the reconstructed trajectories of the gazes and finally the analysis of the focus group transcriptions. The first result was to calculate the number of visits for each exposed item (ROI), as well as the average viewing time for each item. We discovered the items that were most viewed by our participants, such as the letter from the Waffen SS to his wife. Then, we calculated the time spent looking at the different parts that make up the same ROI. This type of calculation revealed for instance a VT discrepancy between the SA soldiers and the Jewish German couple.

Concerning the spatial data provided by eye-tracking, thanks to the coordinates of the fixations and their order provided by the Eye-trackers device, we were able to follow the gaze at several scales, that of the ROI taken in isolation or that of several successive ROIs. We observed how a single ROI could not be separated from the context in which an individual apprehends it and conversely how a singular ROI could influence an individual’s gaze on a group of ROI.

The different results showed: 1. an emotional charge in the eyes of the participants (“E11” gaze trajectory as an example in “Results” section) when reading the letter from the Waffen SS. 2. the gaze of all participants lingering on the ovens and Sonderkommandos depicted in the drawing whilst we note an almost complete absence of fixations on the piles of dead bodies drawn in front of the ovens or at the far end of room. 3. a high density of gazes on the heads of the victims in comparison with the heads of the officers in charge of killing them (see Figure 8B). 4. In order to obtain conclusive observations of the way the participants’ gaze related to different ROIs, we reconstructed global trajectories on a wall of photographs containing several ROIs. The idea was to detect behaviors such as saccades and also to identify the main sub trajectories in the participants gaze paths.

Finally, we performed lexical analysis of the transcripts of the two focus groups that took place before and after the visit to the Caen memorial-museum. This analysis showed that the participants were more eloquent after the visit, they used new words, their knowledge was enriched (assimilation), and some of their patterns were modified to accept new knowledge (accommodation).

### Comparison of the measured VT in our work and the literature:

From our results, we found that the duration of the VT varies among participants. This variability is a notion that has been very present in the literature related to art museum, since manual calculation [21], and even after the use of eye-tracking, studies still reporting this variability [23, 26]. Brieber et al. [23], noted that VT increased with appreciation and understanding of the work, or conditioned by certain properties of stimuli and subjective experiences [48]. Also, certain characteristics such as larger size, complexity, and novelty of the item observed tend to increase VT, for abstract motifs, line drawings, and realistic images [49, 50, 51, 52] as well.

Although in our study, the VT values varied among participants, their average remained high in comparison with the literature. This shows that participants examined the different items with great attention. This result is consistent with those published by Eghbal-Azar & Widlok [ 33].

In this work, we concluded that texts are the most viewed ROI (see Figure 2). In fact, important number of visitors stops to read the texts or the titles of the pictures. This result is somewhat contradictory with the study by Borun & Miller [53], which indicates that visitors read only 18% of the texts in an exhibition, and also the study by Schwan et al. [32] on a similar theme. However, the specific "school" setting of this visit could explain this difference.

Another factor that can affect VT is museum fatigue [54, 23]. Museum fatigue can be caused either by the time spent in the museum itself or the density of the objects on display [55]. This partly explains the lack of success of some sub-areas, such as the Ghetto sub-area or the speed of viewing in the Gypsy sub-area.

### Gaze trajectories and avoidance.

Regarding the spatial data provided by eye-tracking, we studied how visitors looked at a letter written by a Waffen SS to his wife.

The importance of the saccades we noted could indicate a strong emotional charge, especially since the focus groups gathered after the visit seemed to confirm this observation. This remark is in line with that of Smets [56] and Falk & Dierking [2] who think that it is very important to create emotional, sensory, kinesthetic (which concerns the sensation of movement of body parts.), and intellectual experiences, which will be striking for the visitor, in order to allow a good memorization/learning experience.

However, studies by neuroscientists [57] on the relationship between emotion and memory are more nuanced. Starting not from the case of museum visits but from that of PTSD (Post-Traumatic Stress Disorder), they show that if the seat of memory, the hippocampus, is favorably activated by amygdala excitation, the seat of emotions, the mobilization of the amygdala then results in an increasing inhibition of the hippocampus. The debate is important, because it is a question of knowing to what extent emotion can arise interest and therefore learning and if, by mobilizing it too much; the opposite effect is not obtained.

The other relevant result that we have been able to gather from this study is to identify some major mechanisms at work in the visit of history museums. The most striking is undoubtedly that of avoidance. This translates into two behaviors. 1. The refusal to go further in the face of an area of interest that is too violent (see Figure 8.B). 2. Saccades that are characteristic of a strong emotional charge in the face of horror. In fact, according to the results of the fixations (Figure 3), on one of the ROI on this wall, which depicts a group of SA humiliating a woman and her Jewish friend on a street in Cuxhaven, participants read the title, and seem to have looked with more interest at the group of SA on the woman's right side. This result is a priori in contradiction with a more empirical observation which shows that visitors would be more attracted by the "unexpected" [58], in this case the signs around the necks of the two victims, all the more so as the text is written in German and therefore calls for a translation that may be found in the caption, but which here does not exist.

In order to understand these fixations in our corpus, we examined the trajectories of the gazes of the different participants (Figure 6). After applying the density detection algorithm (Figure 7), we noticed that this image is in a very dense sub-path characterized by vertical saccades. Thus, the fixations were generated not by the interest of looking at the SAs but on the contrary by their avoidance. This avoidance is well reinforced by the speech held by all the participants after the visit of the museum, on the executioners (“bourreaux” in French) whose number of occurrences goes from 0 during the focus group before the visit to 23 occurrences after the visit of the memorial-museum (see Figure 9). The participants integrated the notion of the executioner very well from the beginning of the visit by quoting nurses and doctors who had experimented with the disabled, homosexuals, etc. (see Figure 9). In addition, during the visit to the space on proximity extermination (Holocaust by bullets), eye-tracking reported that the participants only looked at the heads and upper bodies of the victims and during the focus group, they said that they consciously avoided looking at the killer soldiers at the mass graves.

This act of avoidance was directed against the soldiers recognized as executioners and not against the barbarity of the act. Indeed, in the photo of the crematory oven (see Figure 5), the participants looked with astonishing precision at the other prisoners putting the dead in the ovens. They detailed all the ovens and described them as "machinery of death," without looking at the pile of dead next to it. For them there was no victim/killer dilemma, all were victims. Participants said that they avoided looking at the killers and only remembered the victims.

This connection between emotion and saccades has already been demonstrated in several studies [19, 59]. The results of these studies showed that positive and negative emotional memories [20] or negative stimuli (e.g. images) triggered more saccades and reduced the duration of fixations.

The result of avoidance was only possible through the use of mobile eye-tracking, which enriches the debate on the comparative merits of mobile and fixed eye-tracking [60]. Both are important but they do not bring the same benefits. The fact that it is possible to work by matching without putting sensors in the track allows the tracking of the routes over a long distance and without impacting on the museum's collections.

### Text-mining of focus groups transcripts:

Thanks to the results of eye tracking, we were able to interpret an avoidance on the other part of the wall of photographs (figures 6 and 7), which contains the photo of the child lying on the ground. This avoidance was detected by recording the vertical saccades. Indeed, the transcription reveals that, prior to the visit; the word child was mentioned when participants were talking about descendants and repairs and/or memory transmission.

After the visit, we note that the use of the word child increased significantly (from 1 to 35 occurrences). The verbatim tells us that the students were shocked by the involvement of children in war, and it was like the first time they were aware of this fact. According to their words, they found it very difficult to integrate this element into a scheme of the war that is predefined in their heads.

### Perspectives and limits:

Our study allowed us to explore a hybrid methodology that combines the use of eye-trackers and focus groups. The particular treatment of spatial data for the reconstruction of gaze trajectories allowed us to detect typical behaviors such as avoidance. However, our approach suffers from two limitations.

The first limitation is the size of the sample. Indeed, it is difficult to generalize the behaviors we found based on such as small number of participants.

The second limitation is the fact that the consolidation of eye-tracking results is based solely on the focus group.

We are aware that both methods have limitations, since participants can be disturbed by the feeling of being observed (eye-tracking), or judged (focus group). In fact, the behavior of students could to become more elaborative than under normal visiting conditions (without eye-tracking).

Also, the scales of some tools used in this study are different, since we used eye-tracking at individual level, and interviews at the group level.

As a perspective, we triggered a more systematic and representative study by recruiting a very large number of participants. In addition to eye-tracking, we are also thinking of introducing detectors for physiological parameters (such as sweating, body movements, etc.) in order to increase the objectivity of our results.

## Ethics and Conflict of Interest

The authors declare that the contents of the article are in agreement with the ethics described in http://biblio.unibe.ch/portale/elibrary/BOP/jemr/ethics.html.

As part of a school outing, after presentation of the project, the teachers were asked to have a parental authorization to the research for each high school student. The authorization covered the protocol with the ASL glasses at the Caen memorial-museum and ensured that the student volunteers would participate in all phases of the research.

The authors declare also that there is no conflict of interest regarding the publication of this paper and that there is no conflict of interest regarding the publication of this paper.

## Acknowledgements

This study was funded by the French Commissariat-General for investment (CGI) via the National Research Agency (ANR) and the “Programme d’investissement pour l’Avenir (PIA)”. The study was realized in the framework of the “équipex” (ANR-EQPX-0021-01) headed by D.P. This program is supported administratively by HESAM Université, bringing together 26 partners (see http://www.matricememory.fr/)

Special thanks go to Stéphane Grimaldi, General Director of the Caen memorial, who hosted and made this research work possible. We would also like to thank Katya Dauchot, Michel Dufossé, Thomas Vallée, Clare Mary Puyfoulhoux who participated to the success of this study and/or providing some ideas in this article. We would like to thank the teaching staff of the high schools Victor Hugo in Caen and Albert Sorel in Honfleur, and the entire MATRICE (“équipex”) team who made this protocol possible.

## References

[b14] Adams, G. D. ( 1983). Museum public relations. American Association for State and Local History.

[b39] Alpern, M. (1969) Types of Movement, H. Davson (Ed.) The Eye. vol.3, 2nd ed, Academic Press, New York.

[b34] ASL MANUAL VERSION 2.02. Eye Tracker Systems Manual. ASL Results & Results-Pro. File Analysis Tool; 2011.

[b22] Batcha, E. , Stein, R. , Filippini-Fantoni, S. , & Leason, T. ( 2012). Evaluating the Practical Applications of Eye Tracking in Museums. Museums and the Web 2012: Proceedings. Archives & Museum Informa-tics.

[b49] Berlyne, D. E. ( 1958). The influence of complexity and novelty in visual figures on orienting responses. Journal of Experimental Psychology, 55, 289–296. 10.1037/h0043555 0022-1015 13513951

[b10] Biran, A. , Poria, Y. , & Oren, G. ( 2011). SOUGHT EXPERIENCES AT (DARK) HERITAGE SITES. Annals of Tourism Research, 38( 3), 820–841. 10.1016/j.annals.2010.12.001 0160-7383

[b54] Bitgood, S. ( 2009). Museum fatigue: A critical review. Visitor Studies, 12, 93–111. 10.1080/10645570903203406 1064-5578

[b53] Borun, M. , & Miller, M. ( 1980). What’s in a name? A study the effectiveness of explanatory labels in a science museum. Franklin Institute.

[b23] Brieber D , Nadal M , Leder H , Rosenberg R (2014). Art in time and space: context modulates the relation between art experience and viewing time. PLoS One; 9(6); 10.1371/journal.pone.0099019. ECollection PMC4043844 24892829

[b50] Brown, L. T. , & Farha, W. ( 1966). Some physical determinants of viewing time under three instructional sets. Perception & Psychophysics, 1, 2–4. 10.3758/BF03207812 0031-5117

[b25] Buquet, C. , Charlier, J. R. , & Paris, V. (1988).Museum application of an eye tracker. Medical & Biomedical Engineering & Computing.,26,277–281. 10.1007/BF02447081 0140-0118 3255017

[b42] Darrien, J. H. , Herd, K. , Starling, L. J. , Rosenberg, J. R. , & Morrison, J. D. (2001).An analysis of the dependence of saccadic latency on target position and target characteristics in human subjects. BMC Neuroscience,2,13. 10.1186/1471-2202-2-13 1471-2202 11696241 PMC59638

[b47] Desbois, P. (2018).The Broad Daylight. The secret Procedures before the Holocaust by Bullets.Skyhorse publishing. Inc.

[b38] Duchowski, A. T. (2003).Eye Tracking Methodology Theory and Practice.Springer-Verlag. 10.1007/978-1-4471-3750-4

[b33] Eghbal-Azar, K. , & Widlok,T. (2012).Potentials and Limitations of Mobile Eye Tracking in Visitor Studies. Social Science Computer Review,31(1),103–118. 10.1177/0894439312453565 0894-4393

[b19] El Haj,M. , Nandrino,J. L. , Antoine,P. , Boucart,M. , & Lenoble,Q. (2017,3).Eye movement during retrieval of emotional autobiographical memories. Acta Psychologica,174,54–58. 10.1016/j.actpsy.2017.02.002 0001-6918 28187309

[b2] Falk,J. H. , & Dierking,L. D. (2013).The Museum Experience Revisited. Measuring the learning impact of museums.Left Coast Press.

[b1] Falk,J. H. , & Dierking,L. D. (2000). Learning from Museums. Visitor Experiences and the Making of Mining. Altamira Press. Chapitre 2. pp. 28-29.

[b24] Filippini-Fantoni,S. , Jaebker,K. , Bauer,D. , & Stofer,K. (2013). Capturing visitors’ gazes. Three eye tracking studies in museums. In The annual conference of museums and the web. 17–20

[b43] Fischer B , Ramsperger E. (1984). Human express saccades: extremely short reaction times of goal directed movements. Exp Brain Res. 57:191-:195. 10.1007/BF00231145 6519226

[b36] Flagg,B. N. (1977). Children and Television: Effects of Stimulus Repetition on Eye Activity. Thesis, Doctor of Education degree, Graduate School of Education, Harvard University;

[b15] Geissler,G. L. , Rucks,C. T. , & Edison,S. W. (2006).Understanding the Role of Service Convenience in Art Museum Marketing: An Exploratory Study.Journal of Hospitality & Leisure Marketing,14(4),69–87. 10.1300/J150v14n04_05 1050-7051

[b26] Heidenreich,S. M. , & Turano,K. A. (2011).Where does one look when viewing artwork in a museum? Empirical Studies of the Arts,29(1),51–72. 10.2190/EM.29.1.d 0276-2374

[b17] Henderson,J. M. , Choi,W. , Lowder,M. W. , & Ferreira,F. (2016).Language structure in the brain: A fixation-related fMRI study of syntactic surprisal in reading. NeuroImage,132,293–300. 10.1016/j.neuroimage.2016.02.050 1053-8119 26908322

[b3] Holmes,J. A. (2011).Informal learning: Student achievement and motivation in science through museum-based learning. Learning Environments Research,14,263– 277. 10.1007/s10984-011-9094-y 1387-1579

[b4] Hooper-Greenhill,E. (2007).Museums and Education: Purpose, Pedagogy, Performance.Routledge. 10.4324/9780203937525

[b11] Isaac,R. K. , Nawijn,J. , van Liempt,A. , & Gridnevskiy,K. (2017).Understanding Dutch visitors’ motivations to concentration camp memorials. Current Issues in Tourism,•••,1–16. 10.1080/13683500.2017.1310190 1368-3500

[b60] Kappoula,Z. , & Lestocart,J. (2018). Esthétique et complexité, vol & Création, expérimentation et neurosciences, idem et Jean-Paul Allouche eds., vol 2 neurosciences, évolution, épistémologie, philosophie, Paris, CNRS Editions

[b37] Karsh,R. , & Breitenbach,F. W. (1983).Looking at looking: The amorphous fixation measure. In R. Groner , C. Menz , D. F. Fisher , & R. A. Monty (Eds.), Eye movements and psychological functions: International views (pp. 53–64).Erlbaum.

[b59] Kaspar,K. , Hloucal,T. M. , Kriz,J. , Canzler,S. , Gameiro,R. R. , Krapp,V. , & König,P. (2013).Emotions’ impact on viewing behavior under natural conditions. PLoS One,8(1),e52737. 10.1371/journal.pone.0052737 1932-6203 23326353 PMC3541363

[b13] Kusenbach,M. (2003).Street phenomenology: The go-along as ethnographic research tool. Ethnography,4(3),455–485. 10.1177/146613810343007 1466-1381

[b35] Lambert,R. H. , Monty,R. A. , & Hall,R. J. (1974).High-speed Data Processing and Unobtrusive Monitoring of Eye Movements. Behavior Research Methods and Instrumentation,6(6),525–530. 10.3758/BF03201340 0005-7878

[b57] Layton,B. , & Krikorian,R. (2002).Memory mechanisms in posttraumatic stress disorder. The Journal of Neuropsychiatry and Clinical Neurosciences,14,254–261. 10.1176/jnp.14.3.254 0895-0172 12154148

[b52] Leckart,B. T. , & Bakan,P. (1965).Complexity judgments of photographs and looking time. Perceptual and Motor Skills,21,16–18. 10.2466/pms.1965.21.1.16 0031-5125 5828372

[b51] Leckart,B. T. (1966).Looking time: The effects of stimulus complexity and familiarity. Perception & Psychophysics,1,142–144. 10.3758/BF03210045 0031-5117

[b48] Leckart,B. T. , & Faw,T. T. (1968).Looking time: A bibliography. Perceptual and Motor Skills,27,91–95. 0031-5125 4879482

[b45] Lee,J. G. , Han,J. , & Whang,K. Y. (2007). Trajectory Clustering: A Partition-and-Group Framework. SIGMOD ’07: Proceedings of the ACM SIGMOD international conference on Management of data;593–604. doi: 10.1145/1247480.1247546

[b16] Loomis,R. J. (1987).Museum visitor evaluation.American Association for State and Local History.

[b46] Loper,E. , & Bird,S. (2002). NLTK: The Natural Language Toolkit. ETMTNLP ’02: Proceedings of the ACL-02 Workshop on Effective tools and methodologies for teaching natural language processing and computational linguistics; 1:63–70. doi: 10.3115/1118108.1118117

[b18] Martinez-Conde,S. , Macknik,S. L. , & Hubel,D. H. (2004).The role of fixational eye movements in visual perception. Nature Reviews. Neuroscience,5(3),229–240. 10.1038/nrn1348 1471-003X 14976522

[b29] Mayr,E. , Knipfer,K. , & Wessel,D. (2009).In-sights into mobile learning: An exploration of mobile eye tracking methodology for learning in museums. In G. Vavoula , N. Pachler , & A. Kukulska-Hulme (Eds.), Researching mobile learning: Frameworks, tools and research designs (pp. 189–204).Peter Lang.

[b55] Melton,A. (1935).Problems of installation in museums of art.American Association of Museums. 10.1037/11526-000

[b27] Milekic,S. (2003).The More You Look the More You Get: Intention-based Interface using Gaze-tracking. In D. Bearman & J. Trant (Eds.), Museums and the Web: Selected papers from Museums and the Web 03.Archives & Museum Informatics

[b28] Milekic,S. (2010). Gaze-Tracking and Museums: Current Research and Implications. In: Archives & Museum Informatics: Museums and the Web 2010, international Conference, Denver, Colorado, USA;

[b7] Nawijn,J. , Isaac,R. K. , Gridnevskiy,K. , & Liempt,A. V. (2015).Holocaust concentration camp memorial sites: An exploratory study into expected emotional response. Current Issues in Tourism,21(2),175–190. 10.1080/13683500.2015.1058343 1368-3500

[b8] Nawijn,J. , Isaac,R. K. , Liempt,A. V. , & Gridnevskiy,K. (2016).Emotion clusters for concentration camp memorials. Annals of Tourism Research,61,244–247. 10.1016/j.annals.2016.09.005 0160-7383

[b9] Nawijn,J. , Isaac,R. K. , Gridnevskiy,K. , & Liempt,A. V. (2018).Holocaust concentration camp memorial sites: An exploratory study into expected emotional response. Current Issues in Tourism,21(2),175–190. 10.1080/13683500.2015.105834 10.1080/13683500.2015.105834 1368-3500

[b20] Nummenmaa,L. , Hyönä,J. , & Calvo,M. G. (2009).Emotional scene content drives the saccade generation system reflexively. Journal of Experimental Psychology. Human Perception and Performance,35(2),305–323. 10.1037/a0013626 0096-1523 19331490

[b6] Packer,J. , Ballantyne,R. , & Bond,N. (2018).Developing an Instrument to Capture Multifaceted Visitor Experiences: The DoVE Adjective Checklist. Visitor Studies,21(2),211–231. 10.1080/10645578.2018.1553925 1064-5578

[b44] R Development Core Team. R: A language and environment for statistical computing. R Foundation for Statistical Computing, Vienna, Austria. 2005; ISBN 3-900051-07-0

[b30] Rainoldi,M. , Neuhofer,B. , & Jooss,M. (2018).Mobile eyetracking of museum learning experiences. In B. Stangl & J. Pesonen (Eds.), Information and Communication Technologies in Tourism (pp. 473–485).Springer.

[b5] Roschelle,J. (1995).Learning in interactive environments: prior knowledge and new experience. In J. H. Falk & L. D. Dierking (Eds.), Public Institutions for Personal Learning (pp. 37–52).American Association of Museums.

[b31] Santini,T. , Brinkmann,H. , Reitstätter,L. , Leder,H. , Rosenberg,R. , Rosenstiel,W. , & Kasneci,E. (2018). The art of pervasive eye tracking: Unconstrained eye tracking in the Austrian Gallery Belvedere. In Proceedings of the 7th workshop on pervasive eye tracking and mobile eye-based interaction. pp. 5:1–5:8. New York:ACM. doi:10.1145/3208031.3208032

[b32] Schwan,S. , Gussmann,M. , Gerjets,P. , Drecoll,A. , & Feiber,A. (2019).Distribution of attention in a gallery segment on the National Socialists’ Führer cult: Diving deeper into visitors’ cognitive exhibition experiences using mobile eye tracking.Museum Management and Curatorship,•••.Advance online publication. 10.1080/09647775.2019.1666422 0964-7775

[b12] Skov,M. , Lykke,M. , & Jantzen,C. (2019).Introducing Walk-Alongs in Visitor Studies: A Mobile Method Approach to Studying User Experience. Visitor Studies,•••,189–210.1064-5578

[b56] Smets,G. (1975).Pleasingness vs interestingness of visual stimuli with controlled complexity: Their relationship to looking time as a function of exposure time. Perceptual and Motor Skills,40,3–7. 10.2466/pms.1975.40.1.3 0031-5125 1118278

[b21] Smith,J. K. , & Smith,L. F. (2001).Spending time on art. Empirical Studies of the Arts,19,229–236. 10.2190/5MQM-59JH-X21R-JN5J 0276-2374

[b58] Soren,B. J. (2009).Museum experiences that change visitors.Museum Management and Curatorship,24(3),233–251. 10.1080/09647770903073060 0964-7775

[b40] Yarbus,A. L. (1967).Eye Movements and Vision.Plenum Press. 10.1007/978-1-4899-5379-7

[b41] Young,L. (1970). Recording eye psition, M. Clynes & M. Milsum (Eds.), Biomedical Engineering Systems, McGraw Hill, New York;

